# Donor age and cold ischemia time combined predict early mortality after long-distance liver procurement

**DOI:** 10.1590/0102-672020260000024e1953

**Published:** 2026-08-17

**Authors:** Agnaldo Soares Lima, Alexandre Imbs Lima, Bruno Ferreira Russo, Bárbara Buitrago Pereira, Eduardo Nacur Silva, Luís Fernando Magalhães Neves, Ana Flávia Passos Ramos, Rafael Rossi Nominato, João Bernardo Sâncio Rocha Rodrigues, José Clóvis Ligeiro, Marcelo de Medeiros Chaves França

**Affiliations:** 1 Santa Casa de Belo Horizonte, Liver Transplant – Belo Horizonte (MG), Brazil.; 2 Universidade Federal de Minas Gerais, Faculdade de Medicina, Postgraduate in Surgery and Ophthalmology – Belo Horizonte (MG), Brazil.; 3 Universidade Professor Edson Antonio Velano – Belo Horizonte (MG), Brazil.

**Keywords:** Liver Transplantation, Cold Ischemia, Donor Selection, Tissue and Organ Procurement, Mortality, Risk Assessment, Transplante de Fígado, Isquemia Fria, Seleção do Doador, Obtenção de Tecidos e Órgãos, Mortalidade, Medição de Risco

## Abstract

**Background:**

The increasing need to expand the donor pool has led to greater utilization of liver grafts procured at a distance from transplant centers, inevitably prolonging cold ischemia time. Whether long-distance procurement independently affects early outcomes or whether risk is primarily driven by donor-related factors remains uncertain.

**Aims:**

We evaluated the impact of procurement distance on early post-transplant survival and investigated whether the combined effect of donor age and cold ischemia time could define a clinically meaningful risk threshold.

**Results:**

We performed a retrospective cohort study of adult liver transplant recipients from March 2016 to December 2025 at a single tertiary transplant center. Donor, recipient, and procurement variables were analyzed according to procurement location. Early mortality was assessed using Kaplan–Meier analysis and Cox proportional hazards modeling. Among 291 transplants, long-distance procurement was associated with significantly longer cold ischemia time but not with increased 15-day mortality. In multivariable analysis, recipient severity (Model for End-stage Liver Disease-Sodium score) and cold ischemia time were independently associated with early mortality, whereas procurement location and donor age alone were not. A composite variable defined by the sum of donor age and cold ischemia time identified a threshold (≥480) associated with significantly reduced early survival, conferring more than a fivefold increase in 15-day mortality independent of procurement distance.

**Conclusions:**

These findings suggest that the risk associated with distant procurement is not geographic but biological, reflecting the interaction between donor susceptibility and ischemic burden. A simple combined metric may help clinicians balance graft-related risks against recipient urgency when evaluating marginal offers.

## ARTICLE HIGHLIGHTS

- Long-distance liver procurement does not compromise transplant outcome.
- Cold ischemia time and donor age impact early mortality after liver transplantation.
- The sum of cold ischemia time and donor age, when greater than 480, represents a higher risk of early mortality after liver transplantation.

## Summary

**CENTRAL MESSAGE:**

Long-distance liver procurement increases cold ischemia time but does not worsen early survival after transplantation. In this study, early mortality was driven by recipient severity and ischemic burden, not by procurement distance itself. A simple combination of donor age and cold ischemia time identified grafts at higher risk of early death. This suggests that risk is not geographic, but biological. A straightforward metric may help clinicians better understand graft quality and guide decision-making when evaluating liver offers.

**PERSPECTIVES:**

Deciding whether to accept a liver graft is often challenging, especially when the organ comes from a distant location. Our findings suggest that distance alone should not discourage acceptance. Instead, clinicians should focus on the interaction between donor age and cold ischemia time. A simple combined metric may provide an intuitive way to assess graft-related risk in real time. This approach could help balance the risks of marginal grafts against the urgency of transplantation, potentially improving decision-making in daily clinical practice.

## VISUAL ABSTRACT

**Figure:**
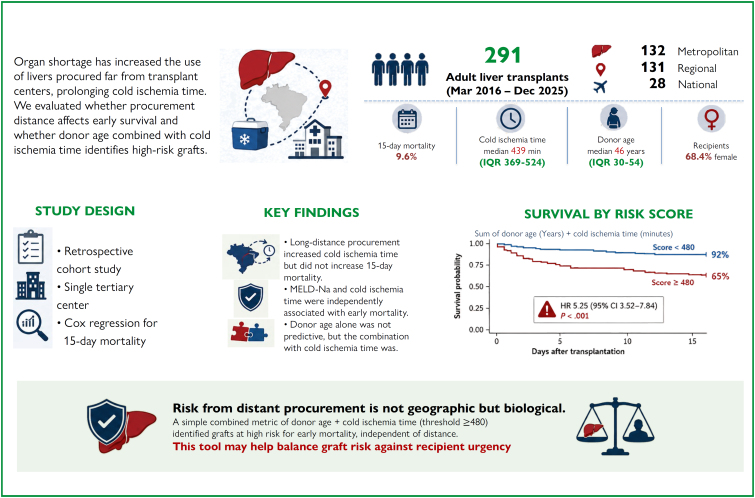

## RESUMO

**Racional:**

A necessidade de expandir o número de doadores motiva equipes a aceitarem enxertos hepáticos captados à distância, prolongando o tempo de isquemia fria. Permanece incerto se captações distantes impactam no resultado do transplante ou se o risco está intrinsecamente relacionado a características dos doadores.

**Objetivos:**

Avaliamos o impacto da distância de captação na sobrevida precoce pós-transplante e investigamos se o efeito combinado da idade do doador e do tempo de isquemia fria poderia definir um limiar de risco clinicamente relevante.

**Resultados:**

Realizado estudo de coorte retrospectiva de 291 receptores adultos de transplante de fígado entre março/2016 e dezembro/2025. Variáveis do doador, do receptor e do processo de retirada-transplante foram analisadas segundo a localidade da captação. A mortalidade precoce foi avaliada pelo método de Kaplan-Meier e modelo de risco proporcional de Cox. Captação em longas distâncias foi associada a tempo de isquemia fria mais longo, sem aumento na mortalidade em 15 dias. Em análise multivariada, a gravidade do receptor (MELD-Na) e o tempo de isquemia fria foram associadas, de maneira independente, com mortalidade precoce, enquanto a localidade da captação e a idade do doador isoladamente, não foram. Uma variável composta, definida pela soma da idade do doador e o tempo de isquemia fria identificou o limite (>480) como associado a redução da sobrevida precoce. Superado tal limite, houve aumento de 5 vezes na mortalidade em 15 dias, independentemente da distância da captação.

**Conclusões:**

Os achados sugerem que o risco associado à captação em longas distâncias não é geográfico, mas biológico, refletindo a interação da suscetibilidade do doador e a agressão isquêmica. Uma combinação métrica simples pode auxiliar no balanço entre risco do enxerto e a urgência em transplantar do receptor, ao avaliar uma oferta de enxerto oriundo de localidade distante.

## INTRODUCTION

The persistent shortage of liver allografts has driven the expansion of donor search strategies, including the use of extended criteria donors and procurement from locations distant from the transplant center.

Expanding donor selection criteria carries an increased risk of inadequate graft function. Several tools have been developed to predict graft dysfunction, although with only moderate sensitivity for adverse outcomes^3,4,7,12^. On the other hand, the use of grafts procured from distant locations inevitably leads to prolonged cold ischemia time (CIT)^8^. CIT remained independently associated with early mortality, reinforcing its central role as a determinant of ischemia-reperfusion injury. The adverse impact of prolonged preservation has been consistently demonstrated across large cohorts and risk modeling studies^4,7^. Experimental and clinical data suggest that ischemic injury is amplified in grafts from older donors, which exhibit reduced metabolic resilience and impaired regenerative capacity^6,13^.

Adequate procurement performance requires correct macroscopic assessment of the graft and careful evaluation of the donor's overall clinical condition^14^. Proper graft perfusion, rapid removal from the abdominal cavity, and preservation of hepatic vascular anatomy are also essential^8^. The impact of macroscopic assessment and graft extraction performed by external teams on post-transplant liver function has been questioned^1,11^.

The decision-making process during organ offers remains a clinical challenge, as surgeons must balance the risks of a suboptimal graft against the urgency of a recipient with a high Model for End-stage Liver Disease-Sodium (MELD-Na) score. Extremes of graft quality — either very good or very poor — facilitate decision-making. More objective references may assist in accepting grafts with unfavorable characteristics but reasonable functional potential.

This study aimed to evaluate the impact of long-distance organ procurement on early post-transplant survival and to identify a clinical risk threshold associated with the combination of donor age and cold ischemia time.

## METHODS

### Study design

This was a single-center retrospective cohort study conducted at a tertiary liver transplantation unit (Santa Casa de Misericórdia de Belo Horizonte, Minas Gerais, Brazil). This study is reported in accordance with the Strengthening the Reporting of Observational Studies in Epidemiology (STROBE) guidelines.

### Study population

We included all adult (≥18 years) liver transplant recipients who underwent transplantation from March 2016 to December 2025. Pediatric and adolescent recipients were excluded because allocation policies and clinical trajectories differ substantially in this population. No other exclusion criteria were applied. The study sample size corresponded to the total number of eligible liver transplants performed during the study period.

### Ethics, consent, and privacy

Ethical approval for this study (CAAE: 82426524.2.0000.5138) was granted by the Ethics Committee Dr. Francisco das Chagas Lima e Silva – Santa Casa de Misericórdia de Belo Horizonte (SCMBH) on October 09, 2024. Due to the retrospective design and no interference with clinical management, an informed consent waiver was approved. Patient identity was protected through data anonymization. All research was conducted in accordance with both the Declarations of Helsinki and Istanbul.

### Data sources and data collection

Recipient and donor clinical and laboratory data were retrieved from the institution's electronic medical record system and complemented with the Brazilian National Transplant System database. Variables were collected for recipients, donors, and procurement logistics, and were recorded using a predefined data abstraction protocol. Data completeness was verified during data abstraction, and variables with incomplete information were tracked and handled according to a prespecified statistical plan.

### Definitions and variables

Recipient characteristics. Recipient sex and age at transplantation were recorded. Indications for transplantation were grouped a priori as:

1. Hepatocellular causes (including alcoholic cirrhosis, metabolic dysfunction–associated fatty liver disease, cryptogenic cirrhosis, hepatitis B and C, among others);
2. Autoimmune diseases (including autoimmune hepatitis, primary sclerosing cholangitis, autoimmune cholangiopathy, primary biliary cholangitis, among others);
3. Urgent indications (including fulminant hepatitis and post-transplant hepatic artery thrombosis); and
4. Miscellaneous indications (including metabolic liver diseases, polycystic liver disease, and Caroli disease, among others). Recipient disease severity was assessed using the MELD-Na score.

Clinical aggravating conditions (special situations). Aggravating conditions were abstracted from clinical records and categorized (e.g., hepatocellular carcinoma, refractory ascites, fulminant hepatitis, and others), as defined in the institutional transplant workflow.

Donor and procurement characteristics. Donor age, sex, body mass index, cause of brain death, aspartate aminotransferase (AST), alanine aminotransferase (ALT), and gamma-glutamyl transferase (GGT) levels, and norepinephrine dose were recorded. CIT was defined (in minutes) as the interval between initiation of cold perfusion in the donor and reperfusion in the recipient. Procurement location was categorized as: metropolitan (local), regional (within the state), and national/interstate (outside the state). The procurement surgeon was classified as belonging to the transplant center team versus an external team.

### Outcomes

The primary outcome was all-cause mortality within 15 days after liver transplantation. The secondary outcomes were characterization of procurement location determinants and the association between procurement location, CIT, and early survival.

### Use of artificial intelligence-assisted tools (transparency statement)

To improve readability, an artificial intelligence-assisted language model (ChatGPT, OpenAI) was used only for language editing, structural alignment of the manuscript text (e.g., clarity, concision, and compliance with journal formatting), and visual abstract production. The tool was not used to generate scientific results, perform statistical analyses, interpret data, or draw conclusions. All content was reviewed and validated by the authors, who take full responsibility for the accuracy and integrity of the manuscript.

### Statistical analysis

General approach. Continuous variables were assessed for distributional characteristics using visual inspection and the Kolmogorov–Smirnov test. Normally distributed variables are presented as mean (standard deviation (SD)); non-normally distributed variables are presented as median (Interquartile Range (IQR)). Categorical variables are presented as n (%).

Group comparisons. Between-group comparisons used Student's t-test/ANOVA for normally distributed continuous variables, and Mann–Whitney/Kruskal–Wallis tests for nonparametric variables, as appropriate. Categorical variables were compared using chi-square or Fisher's exact tests when indicated. Continuous variables were not dichotomized unless clinically justified.

Missing data. Missingness was assessed for all candidate variables before modeling. Among the 291 transplants included in the analysis, information on donor norepinephrine use and dose was unavailable in six cases (2.1%), and the identity of the procurement surgeon (transplant team vs external team) was unavailable in eight cases (2.7%). No other study variables had missing data exceeding 5%.

Given the low proportion of missingness and the absence of a systematic pattern suggesting informative missing data, analyses were conducted using a complete-case approach for each model (listwise deletion). Sensitivity analyses comparing models with and without these variables yielded consistent estimates, supporting the robustness of the primary analysis.

Model building strategy. We conducted analyses with two main objectives:

1. Identify factors associated with 15-day mortality after transplantation; and
2. Characterize procurement location determinants.

Procurement location model. To identify variables independently associated with procurement location, we fitted a multinomial logistic regression model using metropolitan procurement as the reference category. Candidate predictors were restricted to donor/procurement variables (because recipient characteristics do not determine procurement geography) and were selected based on (i) clinical plausibility and (ii) univariable association (p<0.10). To prevent redundancy, variables representing the same logistical construct were not entered simultaneously. Multicollinearity was assessed using variance inflation factors (VIF), with VIF>5 prompting variable removal or recoding.

Early survival model. Early post-transplant survival was analyzed using Kaplan-Meier estimates and compared by the log-rank test. Cox proportional hazards models were used to estimate independent associations with 15-day mortality. The prespecified primary Cox model included MELD-Na, CIT, and procurement location. Additional covariates were considered if clinically relevant and associated with outcome in univariable analyses (p<0.10). Continuous predictors were preferentially modeled as continuous variables; linearity was assessed and, if needed, clinically meaningful transformations or categorized sensitivity analyses were applied.

Composite threshold. A sensitivity analysis evaluated a composite dichotomous exposure defined by the arithmetic sum of donor age (years) and CIT (minutes). The cutoff (≥480) was chosen based on exploratory survival analyses as the value that best separated early survival curves in this cohort. We evaluated candidate cutoffs in increments of 10 and selected the one maximizing log-rank separation while preserving clinical interpretability. Because this is a data-derived threshold, it was treated as hypothesis-generating and interpreted cautiously, with emphasis on the need for external validation.

Model diagnostics and reporting. Proportional hazards assumptions were evaluated using Schoenfeld residuals (or log-minus-log plots) and visual inspection. Model fit was assessed using (i) likelihood ratio tests and (ii) measures such as the Akaike Information Criterion (AIC), for nested comparisons. For the multinomial logistic regression, overall model fit and calibration were assessed using likelihood ratio tests and goodness-of-fit diagnostics appropriate to multinomial models.

Results are reported as hazard ratios (HRs) or odds ratios (ORs), with 95% confidence intervals (CI), and p values, as required.

p values are reported with a leading zero and appropriate rounding conventions (p<0.001 as the smallest reported value).

Software. All analyses were performed using IBM SPSS Statistics, version 22.0.2.0 (IBM Corp., Armonk, NY, USA).

## RESULTS

**Figure 1:**
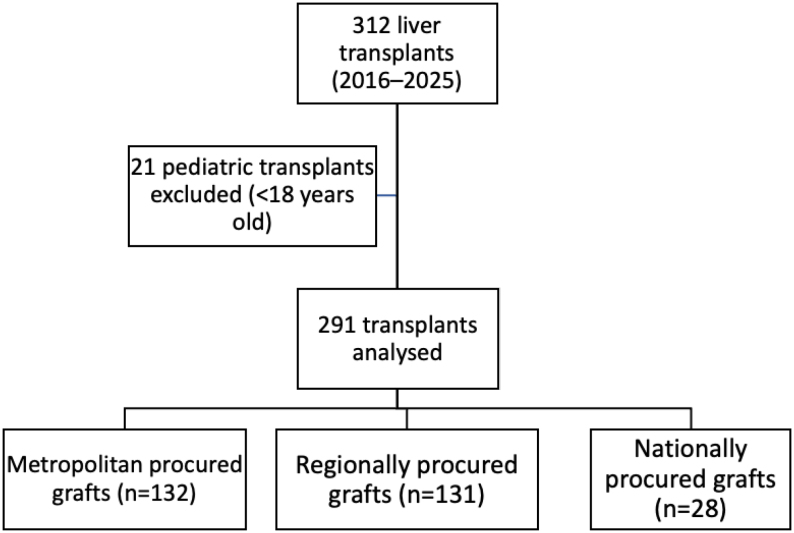
Case selection flowchart.

Of the 312 liver transplants performed between 2016 and 2025, 291 transplants in 275 adult patients were retrospectively selected. Sixteen patients (5.5%) underwent retransplantation. One-year survival was 75.2% for patients and 72.9% for grafts (Figure 1).

Male recipients accounted for 68.4% of cases, with a median age of 58.3 years (IQR 47.5–64.9). In 70.4% of cases, the underlying liver disease was hepatocellular in origin (alcoholic cirrhosis, metabolic dysfunction-associated fatty liver disease, viral or cryptogenic cirrhosis). In 44.3% of cases, the underlying disease was accompanied by aggravating conditions, mainly hepatocellular carcinoma (16.8%) and refractory ascites (15.5%). Other general characteristics of the sample are shown in Table 1.

**Table 1:** Recipient and procurement variables according to 15-day post-transplant survival.

Recipient variables			n	Total sample	15-day survival		p-value
					Yes	No	
Male sex			291	199 (68.4%)	168 (68.9)	31 (66.0%)	0.696
Age, years, median (IQR)			291	58.3 (47.5–64.9)	58.0 (48.0–64.5)	59.1 (43.2–66.1)	0.753
Age categories (years %)	18–40		291	48 (16.5)	39 (16.0)	9 (19.1)	0.629
	41–60			117 (40.2)	101 (41.4)	16 (34.0)	
	+ 60			126 (43.3)	104 (42.6)	22 (46.8)	
Diagnostic groups (%)	Group 1		291	205 (70.4)	177 (72.5)	28 (59.6)	0.062
	Group 2			32 (11.0)	27 (11.1)	5 (10.6)	
	Group 3			19 (6.5)	12 (4.9)	7 (14.9)	
	Group 4			35 (12.0)	28 (11.5)	7 (14.9)	
Clinical complications (%)	No complications of cirrhosis		291	162 (55.7)	136 (84.0)	26 (16.0)	0,061
	HCC			49 (16.8)	41 (38.0)	8 (38.1)	
	Refractory ascites			45 (15.5)	41 (38.0)	4 (19.0)	
	Fulminant hepatitis			13 (4.5)	7 (6.5)	6 (28.6)	
	Others			22 (7.5)	26 (17.5)	3 (14.3)	
MELD-Na, median (IQR %)			290	21.0 (15.0–25.0)	20.0 (15.0–25.0)	24.0 (19.0–29.5)	0.002
**Procurement variables**							
Procurement location (%)		Metropolitan region	291	132 (45.4)	110 (45.1)	22 (46.8)	0.952
		Regional		131 (45.0)	110 (45.1)	21 (44.7)	
		National		28 (9.6)	24 (9.8)	4 (8.5)	
Donor age, years, median (IQR)			291	46.0 (30.0–54.0)	45.0 (29.9–53.6)	48.7 (31.1–54.5)	0.391
Male donor sex (%)			291	183 (62.9)	155 (63.5)	28 (59.6)	0.608
Donor BMI, median, (IQR)			291	24.7 (23.1–26.9)	24.7 (23.1–26.4)	25.7 (23.2–27.6)	0.184
Cause of brain death (%)		Hemorrhagic stroke	291	126 (43.3)	108 (44.3)	18 (38.3)	0.794
		Ischemic stroke		27 (9.3)	21 (8.6)	6 (12.8)	
		Traumatic brain injury		108 (37.1)	89 (36.5)	19 (40.4)	
		Others		30 (10.3)	26 (10.6)	4 (8.5)	
AST U/L (IQR)			291	53.0 (32.0–99.0)	55.0 (32.0–102.0)	49.0 (31.5–90.0)	0.467
ALT U/L (IQR)			291	42.0 (25.0)	47.0 (26.0–96.0)	36.0 (23.0–53.0)	0.036
GGT U/L (IQR)			280	68.0 (28.0–164.0)	71.0 (28.0–171.0)	62.0 (30.5–139.0)	0.641
Norepinephrine >0.10mcg/Kg/min (%)			285	60 (20.6)	51 (21.4)	9 (19.1)	0.726
CIT, minutes, median (IQR)			291	438.8 (368.8–524.3)	436.0 (367.9–507.2)	496.0 (375.3–550.7)	0.069
CIT >480 minutes (%)			291	108 (37.1)	82 (33.6)	26 (55.3)	0.005
Procurement performed by a transplant team surgeon (%)			283	220 (75.6)	184 (77.3)	36 (80.0)	0.691

IQR: interquartile range; HCC: hepatocellular carcinoma; MELD-Na: Model for End-stage Liver Disease-Sodium score – Mann-Whitney and chi-square tests; BMI: body mass index; AST: aspartate aminotransferase; ALT: alanine aminotransferase; GGT: gamma-glutamyl transferase; CIT, cold ischemia time.

Univariable analysis of variables associated with 15-day post-transplant survival is presented in Table 1, which includes recipient-, donor-, and procurement-related characteristics.

Donor biochemical markers (AST, ALT, and GGT) did not demonstrate clinically meaningful differences between survivors and non-survivors, and were not associated with early mortality, despite minor variations observed in univariable comparisons. CIT longer than 480 minutes (eight hours) was associated with higher 15-day mortality (p=0.005). Recipient disease severity, as reflected by the median MELD-Sodium score, was also associated with increased risk of death during this period [20.0 (15.0–25.0) versus 24.0 (19.0–29.5), p=0.002]. Despite longer CIT, transplants performed with grafts procured at a distance from the transplant center did not result in higher mortality. Donor, recipient, and procurement variables were compared across donor procurement locations (Table 2).

**Table 2:** Characteristics according to liver graft procurement location.

Recipient variables		Procurement location			p-value
		Metropolitan region	Regional	National	
15-days survival (%)		83.3	84.0	85.7	0.952
Male sex (%)		94 (71.2)	88 (67.2)	17 (60.7)	0.512
Age, years, median (IQR)		56.7 (47.6–63.2)	59.2 (47.7–66.0)	59.0 (44.2–68.5)	0.273
Age categories (years %)	18–40	24 (18.2)	19 (14.5)	5 (17.9)	0,710
	41–60	56 (42.4)	52 (44.4)	9 (32.1)	
	+ 60	52 (39.4)	60 (45.8)	14 (50.0)	
Diagnostic groups (%)	Group 1	99 (75.0)	88 (67.2)	18 (64.3)	0,314
	Group 2	11 (8.3)	18 (13.7)	3 (10.7)	
	Group 3	9 (6.8)	6 (4.6)	4 (14.3)	
	Group 4	13 (9.8)	19 (14.5)	3 (10.7)	
Clinical complications (%)	HCC	20 (36.4)	26 (41.9)	3 (25.0)	0,054
	Refractory ascites	21 (38.2)	21 (33.9)	3 (25.0)	
	Fulminant hepatitis	5 (9.1)	6 (9.7)	2 (16.7)	
	Others	9 (16.3)	9 (14.5)	4 (33.3)	
MELD-Na, median (IQR)		21.0 (16.0–25.0)	20.0 (15.0–26.0)	22.0 (15.0–29.0)	0.638
**Procurement variables**					
Donor age, years, median (IQR)		50.0 (38.2–56.2)	40.4 (26.9–52.9)	37.9 (25.6–56.4)	<0.001
Male donor sex		73 (55.3)	95 (72.5)	15 (8.2)	0.009
Donor BMI, median, (IQR %)		25.1 (23.3–27.5)	24.7 (23.1–26.4)	23.9 (22.6–26.8)	0.305
Cause of brain death (%)	Hemorrhagic stroke	69 (52.3)	50 (38.2)	7 (25.0)	0,004
	Ischemic stroke	13 (9.8)	9 (6.9)	5 (17.9)	
	Traumatic brain injury	34 (25.8)	62 (47.3)	12 (42.9)	
	Others	16 (12.1)	10 (7.6)	4 (14.2)	
AST U/L (IQR)		52.0 (28.0–88.0)	55.5 (33.0–138.3)	53.0 (33.0–116.0)	0.169
ALT U/L (IQR)		36.0 (25.0–63.0)	53.5 (25.0–125.0)	48.0 (31.0–115.0)	0.016
GGT U/L (IQR)		60.0 (28.0–132.0)	74.0 (29.0–214.8)	62.0 (24.0–125.0)	0.144
Norepinephrine >0.10mcg/Kg/min (%)		25 (19.4)	28 (21.9)	7 (25.0)	0.767
CIT, minutes, median (IQR)		381.4 (339.9–445.4)	475.4 (415.5–533.0)	606.2 (540.2–667.0)	<0.001
CIT >480 minutes (%)		20 (15.2)	64 (48.9)	24 (85.7)	<0.001
Procurement performed by a transplant team surgeon (%)		121 (94.5)	86 (67.2)	13 (48.1)	<0.001

IQR: interquartile range; HCC: hepatocellular carcinoma; MELD-Na: Model for End-stage Liver Disease-Sodium score – Kruskal-Wallis and χ^2^ tests; BMI: body mass index; AST: aspartate aminotransferase; ALT: alanine aminotransferase; GGT: gamma-glutamyl transferase; CIT: cold ischemia time.

**Figure 2:**
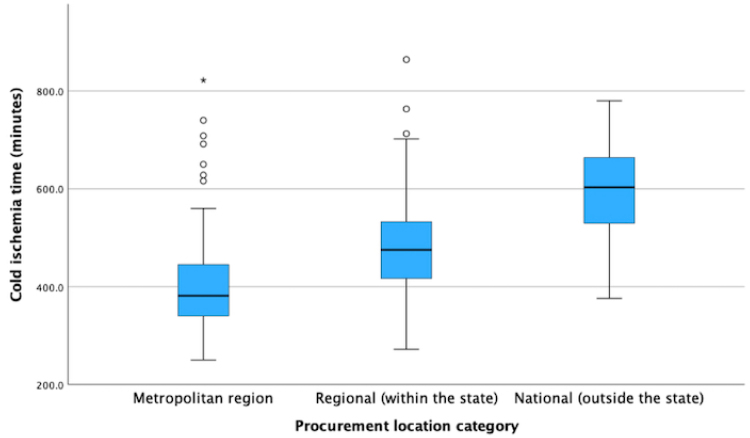
Distribution of cold ischemia time according to liver graft procurement location.

Procurement location was associated with CIT duration (p<0.001) (Figure 2).

Variables showing statistically significant differences across procurement locations were included in a multinomial logistic regression model, which demonstrated that CIT, donor age, and procurement performed by an external team were independently associated with procurement outside the Metropolitan Region. Recipient-related variables did not differ among groups, indicating that long-distance procurement was primarily determined by logistical factors and donor profile. While prolonged CIT and procurement by external teams are inevitable consequences of increased distance from the transplant center, younger donor age appears to reflect selective donor choice.

We then evaluated whether these procurement-related differences translated into differences in early post-transplant survival. Cox proportional hazards models were constructed to assess the impact of CIT and donor risk combined on 15-day post-transplant survival (Table 3). Procurement location, MELD-Sodium score, donor age, CIT, and the interaction between donor age and CIT were considered. The interaction term did not remain significant and was not included in the final model.

**Table 3:** Cox regression models for mortality within 15 days after transplantation.

Variable	HR	95%CI	p-value
Primary model (continuous variables)			
Recipient severity			
MELD-Na score (per point)	1.06	1.03–1.10	<0.001
Graft characteristics/logistics			
Cold ischemia time (per hour)	1.25	1.07–1.46	0.005
Donor age (per year)	1.00	0.98–1.03	0.748
Procurement location			
Metropolitan region	Reference	—	—
Regional	1.21	0.74–1.99	0.450
National	1.33	0.71–2.50	0.370
Sensitivity analysis (graft risk threshold)			
Sum (donor age + CIT) ≥ 480	5.25	3.52–7.84	<0.001
MELD-Na score (per point)	1.05	1.02–1.09	0.002
Procurement location	Not significant	—	>0.05

HR: hazard ratio; 95%CI: confidence interval; MELD-Na score: Model for End-stage Liver Disease-Sodium score; CIT: cold ischemia time (expressed in minutes for the composite variable).

**Figure 3:**
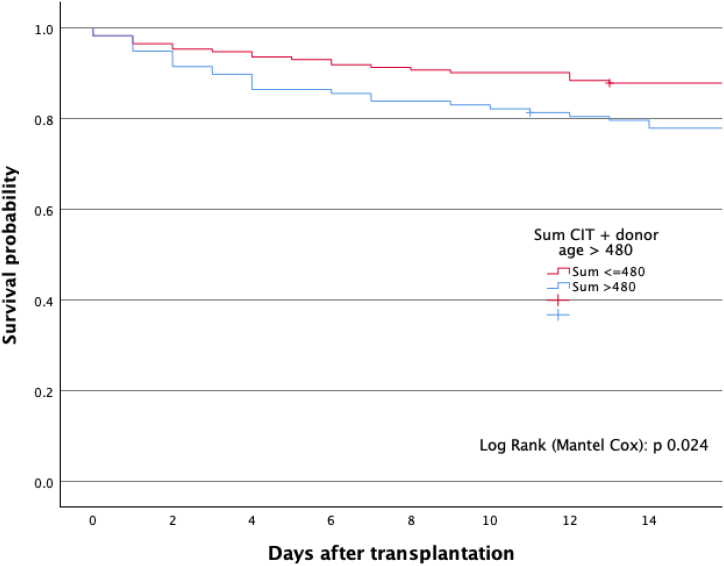
Post-transplantation survival according to donor age and cold ischemia time combined.

After adjustment, there was no evidence of increased risk associated with procurement outside the Metropolitan Region; conversely, the "national" group showed a hazard ratio below one, likely reflecting donor selection. The MELD-Sodium score remained strongly and independently associated with early mortality (HR 1.07 per point; 95%CI 1.035–1.107; p<0.001). CIT also remained independently associated with early mortality (HR 1.316 per hour; 95%CI 1.123–1.542; p<0.001). Donor age alone was not associated with 15-day mortality. A secondary analysis of the combined donor age and CIT interaction demonstrated that, at the cutoff value of 480, survival curves differed significantly (log-rank χ^2^=6.075; p=0.014). Patients receiving grafts in which the combination of donor age and CIT exceeded this threshold had more than a fivefold higher risk of 15-day mortality, independent of procurement location (Figure 3).

## DISCUSSION

The growing reliance on organs procured from outside the local donation area reflects an unavoidable response to the persistent imbalance between organ supply and demand. Recent reports have shown that broader sharing and long-distance procurement can safely expand access to transplantation when logistical processes are optimized^8^. However, concerns remain that prolonged preservation time and variability in procurement practices may negatively influence graft function.

In the present study, procurement distance itself was not associated with early mortality, despite significantly longer CITs. This finding supports the concept that geographic distance is not intrinsically harmful but instead acts as a surrogate for ischemic exposure and donor selection. Similar observations have been reported in analyses of long-distance graft utilization, in which outcomes were largely determined by donor and preservation factors rather than transport logistics alone^8^.

Donor age alone was not independently associated with mortality in our analysis, but its interaction with ischemic duration proved clinically meaningful. Prior investigations have shown that the cumulative effect of donor age and CIT results in inferior graft survival compared with either factor alone, emphasizing the importance of evaluating these variables jointly rather than independently^13^. This biological interaction likely reflects diminished tolerance to oxidative stress and microcirculatory dysfunction in aging grafts. We did not observe a consistent association between donor transaminases and early mortality, suggesting that routine biochemical injury markers may be less informative than donor age and ischemic burden in this context.

Risk prediction models such as the Donor Risk Index and D-MELD (donor risk and Model for End-Stage Liver Disease) have attempted to quantify donor-recipient matching but remain limited in bedside applicability during real-time organ acceptance^2,4,5,7,9^. Our findings extend this body of work by proposing a simplified composite parameter that integrates donor susceptibility and preservation injury into a single, intuitive metric that may assist clinical decision-making.

Importantly, the identified threshold should not be interpreted as an absolute contraindication to graft utilization. Transplant benefit must always be considered in relation to recipient urgency. The MELD system was originally developed to predict waitlist mortality, and in high-risk candidates, accepting a marginal graft may still confer a net survival advantage^10^. Thus, the combined donor age–ischemia metric may function best as a contextual tool to inform risk–benefit assessment rather than as a rigid allocation rule.

The absence of worse outcomes in distant procurements in this cohort likely reflects adaptive donor selection, with younger grafts preferentially accepted when longer preservation times are anticipated. Such behavior highlights how transplant teams dynamically mitigate risk even without formalized thresholds.

This study has several limitations inherent to its retrospective, single-center design. Although consecutive cases were included to minimize selection bias, unmeasured confounding cannot be excluded. Decisions regarding organ acceptance were made in real time by the transplant team and may have introduced center-specific selection practices that are not fully captured by recorded variables.

Granular markers of graft quality, such as histologic steatosis assessment and detailed donor hemodynamic data, were not consistently available and therefore could not be incorporated into multivariable modeling. Similarly, donor biochemical parameters (AST, ALT, and GGT) did not demonstrate consistent associations with early mortality in this cohort and were treated as descriptive variables rather than predictors.

The proposed composite threshold combining donor age and CIT was derived from observational data within this dataset and should be considered hypothesis-generating. Because this cutoff was not externally validated, its clinical applicability requires confirmation in independent populations with different allocation dynamics.

Finally, the study evaluated very early mortality (15 days), a time frame selected to capture events most directly related to preservation injury and perioperative graft dysfunction. Longer-term outcomes were not modeled and may be influenced by additional recipient- and immunologic-related factors beyond the scope of this analysis.

## CONCLUSIONS

Long-distance liver procurement can be performed without compromising early outcomes when donor biological reserve and ischemic burden remain balanced. Integrating donor age and CIT into a unified framework may provide a pragmatic approach to risk stratification in an era increasingly dependent on extended-criteria grafts.

## Data Availability

The datasets generated and/or analyzed during the current study are available from the corresponding author upon reasonable request.
